# Magneto-optical painting of heat current

**DOI:** 10.1038/s41467-019-13799-7

**Published:** 2020-01-07

**Authors:** Jian Wang, Yukiko K. Takahashi, Ken-ichi Uchida

**Affiliations:** 1 National Institute for Materials Science, Tsukuba, 305-0047 Japan; 2 Center for Spintronics Research Network, Tohoku University, Sendai, 980-8577 Japan; 3 Department of Mechanical Engineering, The University of Tokyo, Tokyo, 113-8656 Japan

**Keywords:** Thermoelectric devices and materials, Spintronics, Magneto-optics, Electronic and spintronic devices

## Abstract

Active control of heat flow is crucial for the thermal management of increasingly complex electronic and spintronic devices. In addition to conventional heat transport engineering, spin caloritronics has received extensive attention as a heat control principle owing to its high controllability and unique thermal energy conversion symmetry. Here we demonstrate that the direction of heat currents generated by spin-caloritronic phenomena can be changed simply by illuminating magnetic materials with visible light. The optical control of heat currents is realized through a combination of the spin-driven thermoelectric conversion called an anomalous Ettingshausen effect and all-optical helicity-dependent switching of magnetization. This approach enables not only pinpoint manipulation and flexible design of the heat current distribution by patterning the illuminating light but also on/off control of the resulting temperature modulation by tuning the light polarization. These versatile heat control functionalities will open up a pathway for nanoscale thermal energy engineering.

## Abstract (web summary)

Spin caloritronics offers advantages for the thermal management of spintronic devices. Here, the authors demonstrate that the direction of heat currents generated by spin-caloritronic phenomena can be changed by illuminating magnetic materials with visible light.

## Introduction

**Fig. 1:**
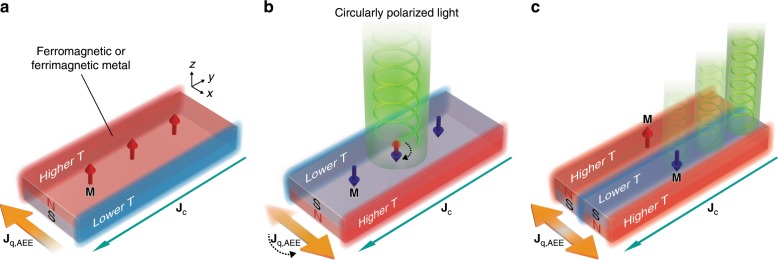
Concept of magneto-optical painting of heat current. **a** Schematic of the anomalous Ettingshausen effect (AEE). When a charge current **J**_c_ passes through a ferromagnetic or ferrimagnetic material with the magnetization vector **M**, the AEE creates a heat current **J**_q,AEE_ in the direction of the cross product of **J**_c_ and **M** (Eq. (1)). **b** Light-induced reversal of the AEE-induced heat current. When illuminating the material with circularly polarized light, the **M** direction is reversed via the all-optical helicity-dependent switching (AO-HDS). Then, the **J**_q,AEE_ direction is also reversed following the AEE symmetry. **c** Magneto-optical design of the heat-current distribution. The local **J**_q,AEE_ direction and resulting temperature modulation distribution can be controlled with magnetic domains pre-designed via the AO-HDS.

Spin caloritronics is the emerging field that combines the spin degree of freedom with thermal transport and thermoelectric conversion^1,2^. Recent experiments on spin caloritronics have resulted in significant progresses in fundamental studies on various heat-current-generation phenomena driven by charge and/or spin currents^3–9^, which can modulate the temperature of magnetic materials and junctions, depending on the direction of spins. The anomalous Ettingshausen effect (AEE)^6–12^, one of such phenomena in magnetic materials, refers to the generation of a heat current **J**_q,AEE_ in the direction of the cross product of a charge current **J**_c_ and magnetization **M** (Fig. 1a):

1 $${\mathbf{J}}_{{\mathrm{q,AEE}}} \propto {\mathbf{J}}_{\mathrm{c}} \times {\mathbf{M}}.$$

The rapid developments of spin caloritronics have led recently to detailed studies on the AEE and its observation in thin films^6,8,9^. The symmetry of the AEE enables reversible manipulation of the **J**_q,AEE_ direction by tuning the **M** direction; such magnetic control of heat currents was impossible with conventional thermoelectrics alone.

In this work, we propose and demonstrate active control of the AEE-induced heat current by illuminating magnetic materials with visible light. The optical control of the heat current is realized by combining the AEE with all-optical helicity-dependent switching (AO-HDS) of magnetization^13–21^. The AO-HDS refers to deterministic magnetization switching in magnetic materials as a result of circularly polarized light illumination, where the orientation of **M** is determined by the light helicity. The **M** reversal due to the AO-HDS can be achieved by using femtosecond circularly polarized light without applying external magnetic fields. Although the AEE and AO-HDS have been investigated independently so far, we find that simultaneous use of these phenomena enables the optical control of the heat current. As shown in Fig. 1b, when a magnetic material exhibiting the AEE and AO-HDS is illuminated with circularly polarized light and a charge current is applied to the material, the direction of **J**_q,AEE_ is reversed in response to the light-induced **M** reversal according to Eq. (1). Importantly, the **J**_q,AEE_ reversal due to the AO-HDS appears only in the illuminated area; by patterning the illuminating light, the **J**_q,AEE_ distribution and, thereby, the resulting temperature distribution can be freely tailored (Fig. 1c). The local manipulation and flexible design of the heat-current distribution are exclusive features of spin-driven thermoelectric phenomena combined with the AO-HDS, providing active thermal management principles for electronic and spintronic devices. The concept of the magneto-optical painting of heat currents can be extended to various spin-caloritronic phenomena, such as the spin Peltier effect^3,5^ and the anisotropic magneto-Peltier effect^4,7,9^, while we focus on the AEE for the proof-of-concept in this work.

## Results

### Magneto-optical properties

**Fig. 2:**
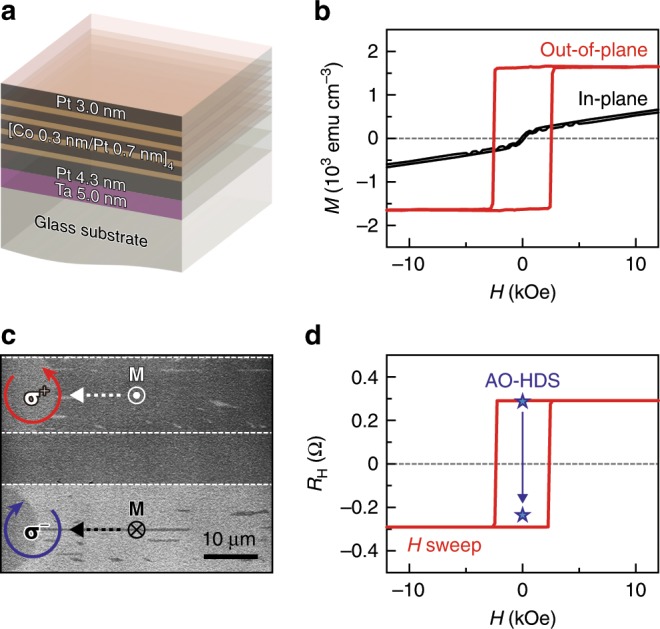
Magnetic and magneto-optical properties of Co/Pt multilayer. **a** Layer configuration of the [Co/Pt]_4_ multilayer film. **b** Magnetization *M* curves of the [Co/Pt]_4_ sample, measured when the magnetic field *H* was applied along the easy axis (out-of-plane direction of the film) and the hard axis (in-plane direction of the film) at room temperature. The [Co/Pt]_4_ sample exhibits the strong perpendicular magnetic anisotropy with an out-of-plane coercive field of 2.3 kOe and an anisotropy field of 41.9 kOe. **c** Magneto-optical Kerr effect microscopy image of the [Co/Pt]_4_ sample illuminated with right (**σ**^+^) and left (**σ**^−^) circularly polarized light at room temperature. The dark (bright) contrast represents the area with **M** along the upward (downward) direction perpendicular to the film plane. **d** Hall resistance *R*_H_ of the [Co/Pt]_4_ sample with a Hall cross shape at room temperature. The red line shows the out-of-plane *H* dependence of *R*_H_. The blue star data points were measured before and after illuminating the whole Hall cross with **σ**^−^ light at zero field, where the cross was uniformly magnetized by an external field of *H* = + 20 kOe before the light illumination.

To demonstrate the magneto-optical control of the AEE-induced heat current, we used alternately stacked [Co/Pt]_*n*_ multilayer films, which are known to exhibit the AO-HDS of magnetization^16,20^. We optimized the thickness of the Co and Pt layers and the Co/Pt bilayer number *n* for the deterministic AO-HDS, as shown in Fig. 2a (see also Methods and Supplementary Fig. 1). Figure 2b shows the magnetization curves of the optimized [Co/Pt]_4_ sample at room temperature; the multilayer film was observed to possess strong perpendicular magnetic anisotropy. In order to prove the deterministic AO-HDS, a magneto-optical Kerr effect microscopy image of the [Co/Pt]_4_ sample was recorded after parts of the sample were illuminated with right (**σ**^+^) and left (**σ**^−^) circularly polarized light (Fig. 2c). The dark background of the magneto-optical image represents the magnetic domain with the magnetic moments pointing out of the film plane, since the sample was initially magnetized along the upward direction. While the dark contrast is barely changed after sweeping with **σ**^+^ light, the conversion from the dark to bright was observed after sweeping with **σ**^−^ light, indicating that the [Co/Pt]_4_ sample exhibits the clear AO-HDS of magnetization. Our Hall resistance measurements show that ~91% of the magnetic moments are reversed by illuminating the [Co/Pt]_4_ sample with the circularly polarized light (Fig. 2d; Methods). Similar results were obtained also for the [Co/Pt]_3_ and [Co/Pt]_5_ samples with the same Co and Pt thicknesses. This high AO-HDS efficiency confirms that the [Co/Pt]_*n*_ films are a good platform for demonstrating the magneto-optical control of the AEE-induced heat current.

### Thermal imaging of AEE

**Fig. 3:**
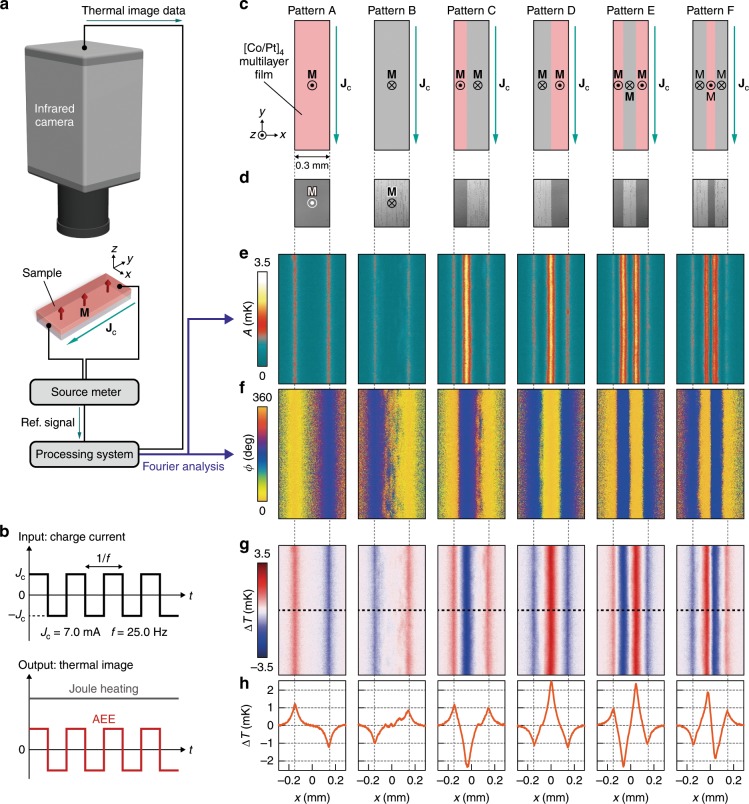
Demonstration of magneto-optical painting of heat current. **a** Lock-in thermography method for the AEE measurements. The temperature modulation synchronized with the charge current is extracted by Fourier analysis. **b** Time charts of the input charge current and output temperature changes induced by the AEE and Joule heating. In the AEE measurements, the square-wave-modulated alternating charge current with the amplitude *J*_c_ = 7.0 mA, frequency *f* *=* 25.0 Hz, and zero offset was applied to the samples. **c** Magnetic domain patterns A–F designed via the AO-HDS on the [Co/Pt]_4_ strips. **d** Magneto-optical images corresponding to the patterns A–F. **e**, **f** Lock-in amplitude *A* (**e**) and phase *ϕ* (**f**) images for the [Co/Pt]_4_ strips with the patterns A–F. **g** *∆T* (= *A*cos*ϕ*) images for the [Co/Pt]_4_ strips. Each image consists of 192 × 370 pixels. **h** Surface *∆T* profiles along the *x* direction. The profiles were obtained by averaging 50 *x*-directional raw profiles along the *y* direction; the center of the averaged area is marked with black dotted lines in **g**.

The temperature change induced by the AEE in the [Co/Pt]_*n*_ films was visualized at room temperature by means of the lock-in thermography technique^5–9,22^ (Fig. 3a). Here, we measured the spatial distribution of infrared radiation thermally emitted from the sample surface while applying a square-wave-modulated alternating charge current with the amplitude 7.0 mA, frequency *f* = 25 Hz, and zero offset to the strip-shaped [Co/Pt]_*n*_ sample. The AEE-induced temperature modulation due to such an alternating charge current oscillates with *f* since the AEE responds linearly to the current, while the concomitant Joule-heating background is constant over time (Fig. 3b)^6–9^. Therefore, by extracting the first harmonic response of the detected thermal images, the pure AEE contribution free from the background can be obtained. The first harmonic contribution is converted into the lock-in amplitude *A* and phase *ϕ* images through the procedure detailed in the Methods section. All the lock-in thermography measurements were performed in the absence of external magnetic fields. Owing to the strong perpendicular magnetic anisotropy of our [Co/Pt]_*n*_ films, magnetic domain patterns pre-designed via the AO-HDS are preserved during the lock-in thermography measurements, where the magnetization of the films is in the upward or downward direction. It should be noted that, since the magnetic structure of the films reaches a steady state before the AEE measurements, light-induced ultrafast spin dynamics and resulting nonequilibrium thermo-spin effects^23^ do not affect the thermal images presented in this study. Under this condition, the AEE contribution can be distinguished from possible contributions coming from the spin Peltier effect and the anisotropic magneto-Peltier effect, which exhibit different symmetries in the heat-current generation. As the temperature modulation induced by the spin Peltier effect appears only in an in-plane magnetized configuration^6^, its contribution is eliminated in our perpendicularly magnetized films. Then, the temperature modulation induced by the anisotropic magneto-Peltier effect in the strip-shaped sample can appear only when the charge current flows across magnetic domain boundaries with nonuniform **M** distributions^7^. This behavior is completely different from the AEE-induced temperature modulation, and the contribution of the anisotropic magneto-Peltier effect can be neglected in our experiments because the domain wall size (<100 nm)^20^ is much smaller than the spatial resolution of our infrared camera (~10 μm).

The leftmost *A* and *ϕ* images in Fig. 3e, f are the results of the lock-in thermography measurements for the [Co/Pt]_4_ strip uniformly magnetized along the +*z* direction (Pattern A). The *A* image shows that a clear temperature modulation appears around the edges of the [Co/Pt]_4_ strip, and the *ϕ* image shows that the sign of the temperature modulation at the right edge is opposite to that at the left edge because of the *ϕ* difference of ~180°. These results indicate that the heat current is generated along the *x* direction in the [Co/Pt]_4_ strip by applying the charge current along the *y* direction, consistent with the orthogonal relation between the heat and charge currents for the AEE in a perpendicularly magnetized ferromagnet (Eq. (1))^6,8^; this result confirms that magneto-optical materials exhibit the AEE. Hereafter, to provide an intuitive understanding of the experimental results, we discuss the behaviors of the AEE in terms of *∆T* (= *A*cos*ϕ*), where *∆T* is the current-induced temperature modulation, including sign information. The leftmost *∆T* image and profile in Fig. 3g, h show that the temperature near the right (left) edge of the [Co/Pt]_4_ strip decreases (increases) due to the AEE.

### Magneto-optical control of AEE-induced heat current

**Fig. 4:**
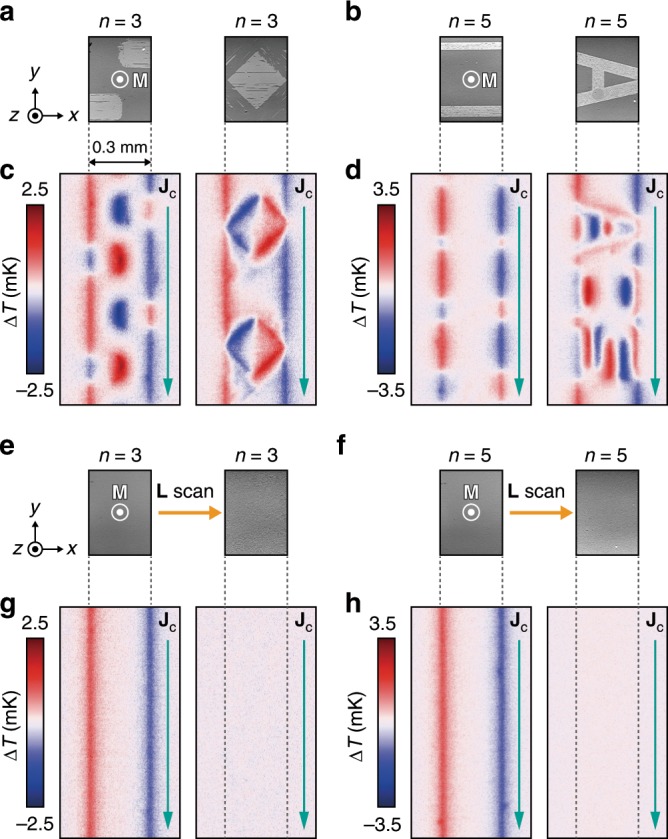
Flexible design and on/off control of temperature modulation. **a**, **b** Magneto-optical images for the [Co/Pt]_3_ (**a**) and [Co/Pt]_5_ (**b**) strips with pre-designed magnetic domain patterns. The left image in **a** shows the magnetic domains with periodical rounded rectangles that start from the edges and terminate at the middle of the strip, while the right image shows diamond-shaped patterns. The left image in **b** shows line patterns with different widths, while the right image shows the alphabet patterns AOS, where only the character A is presented in the viewing area of the microscope. All the domain patterns were prepared by means of the AO-HDS. **c**, **d** Corresponding *∆T* images for the [Co/Pt]_3_ (**c**) and [Co/Pt]_5_ (**d**) strips. The difference in the magnitude of the *∆T* signals between the [Co/Pt]_3_ and [Co/Pt]_5_ samples can be explained by the thickness dependence of the AEE-induced temperature modulation^9^. **e**, **f** Magneto-optical images for the [Co/Pt]_3_ (**e**) and [Co/Pt]_5_ (**f**) strips before and after linearly polarized light (**L**) illumination. **g**, **h** Corresponding *∆T* images for the [Co/Pt]_3_ (**g**) and [Co/Pt]_5_ (**h**) strips. The AEE-induced temperature modulation is macroscopically canceled out after **L** illumination due to the creation of random multiple domains.

To demonstrate the magneto-optical painting of the AEE-induced heat current, magnetic domain patterns with various configurations were designed via the AO-HDS using the [Co/Pt]_4_ strips (see the patterns B-F in Fig. 3c). Figure 3d shows the magneto-optical images for each domain pattern, where the dark- and bright-contrast areas represent the magnetic domains with **M** ||+*z* and **M** || −*z*, respectively, and the strips were initially magnetized along the + *z* direction before the pattering. The pattern B consists of a single magnetic domain in which the magnetization of the [Co/Pt]_4_ strip is uniformly reversed by the AO-HDS, while the patterns C–F consist of optically painted multiple domains with a striped shape. By comparing the *∆T* images between the patterns A and B, we found that the sign of *∆T* around the edges of the [Co/Pt]_4_ strip is reversed as a consequence of the uniform **M** reversal due to the AO-HDS, indicating the clear directional control of the AEE-induced heat current by circularly polarized light (Fig. 3g, h). In addition to the uniform heat-current reversal, we also demonstrated the local manipulation of the heat-current direction by using the other pre-designed domain patterns. The *∆T* images for the patterns C–F show that multiple cooling and heating signals appear alternately along the *x* direction, where the temperature profile along the *y* direction remains unchanged (Fig. 3g). These *∆T* distributions can be explained by the fact that the AEE generates a heat current in each magnetic domain following Eq. (1), and the temperature modulation appears as a result of the superposition of the contributions stemming from the optically painted domain patterns. Importantly, although the AEE in a uniformly magnetized ferromagnet can modulate the temperature only around the edges of the strip, the combination of the AEE and AO-HDS allows one to modulate the temperature at arbitrary positions in the sample simply by designing magnetic domain patterns, which can act as a principle for local cooling and heating. The magnitude of the temperature modulation at the domain boundaries shows a twofold increase owing to the concentration of the heat currents from the adjacent domains (Fig. 3g, h), providing a valid approach to enhance the AEE-induced temperature modulation. These behaviors were observed also for the [Co/Pt]_*n*_ multilayer films with different *n* values (Fig. 4) as well as ferrimagnetic Fe_72_Tb_28_ films exhibiting the AO-HDS^14^ (Supplementary Fig. 2), confirming the generality of this method. Furthermore, the versatility of the optical control method for the AEE-induced heat current is demonstrated with more complicated magnetic domain patterns; as shown in Fig. 4a–d, the distribution of the AEE-induced temperature modulation can be freely programed following the pre-designed magnetic domain patterns. With the simple setup used in this study, the spatial resolution limit of this magneto-optical heat-current control is determined by the wavelength of the applied laser light. By combining this approach with plasmonics, near-field optics^24^, and/or structural geometry design^17^, the spatial resolution can further be improved, enabling precise and local thermoelectric cooling and heating of nanoscale devices.

The AO-HDS enables not only the directional manipulation of the AEE-induced heat current but also on/off control of the resulting temperature change. We found that the AEE-induced temperature modulation can be switched off by illuminating magnetic materials with linearly polarized light (**L**), instead of circularly polarized light. Since the **L** illumination creates multiple domains with random patterns and equal populations of the upward and downward magnetic moments (Fig. 4e, f), the AEE-induced heat currents are macroscopically canceled out. We demonstrated this feature by using the same [Co/Pt]_*n*_ strips. In Fig. 4g, h, we compare the *∆T* images for the [Co/Pt]_*n*_ samples before and after the **L** illumination, where the samples were uniformly magnetized along the +*z* direction before the illumination. The comparison shows that, while the clear AEE-induced temperature modulation appears in the uniformly magnetized [Co/Pt]_*n*_ strips, it disappears after the **L** illumination (note that the size of each domain after the illumination is much smaller than the spatial resolution of the infrared camera). The AEE-induced temperature modulation can be switched on again by illuminating the samples with circularly polarized light, since the AO-HDS of magnetization is a reversible process among right circularly, left circularly, and linearly polarized light.

## Discussion

The magneto-optical control of heat currents demonstrated here can be applied directly to versatile nano-thermal controllers that enable pinpoint temperature modulation in electronic and spintronic devices. One of the remarkable features of this technique is that the cooling and heating properties can be designed flexibly in a reconfigurable and programmable manner, since the heat current can be manipulated via the light helicity and illumination patterns. Significantly, the spatial resolution of the heat-current control can reach the diffraction limit of the laser light used for the AO-HDS, which can further be improved by confining light energy in nanoscale through the smart engineering techniques^17,24^. Although this work focuses on the steady-state properties, the investigation of nonequilibrium heat-current-generation properties due to light-induced ultrafast spin dynamics can be one of the future works. It is also worth to notice that the magnitude and temporal response of the AEE-induced temperature modulation observed here are affected by the heat transfer from the film sample to the substrate^6,8,9^; if the heat loss to the substrate is suppressed, the AEE should exhibit much larger temperature modulation with much faster response. Therefore, the optimization of the thermal design of AEE-based devices is necessary toward their practical use, although the signal-to-noise ratio and temporal response obtained in our experiments are good enough for the proof-of-concept.

Finally, we would like to emphasize the versatility of the magneto-optical heat control technique. The proposed method should be valid for various magneto-optical materials exhibiting the AO-HDS^13–21,25^, including not only ferromagnets and ferrimagnets but also antiferromagnets and synthetic ferrimagnets which cover metals, oxides, alloys, multilayers, and heterostructures. Potentially, the effect is generalizable to all magnetic materials through exchange coupling with magneto-optical materials^19,25^, holding the promise of highly efficient optical heat-current control with the use of materials showing large AEE. Here, the improvement of the thermoelectric conversion efficiency based on the AEE can be realized, for example, by utilizing topological materials^26^ and interface effects^27,28^ and by optimizing multilayer structures^27,28^. In addition to the AEE in perpendicularly magnetized materials, the magneto-optical painting of heat currents can be realized by various spin-caloritronic phenomena, including the spin Peltier effect and the anisotropic magneto-Peltier effect, if in-plane magnetized materials exhibiting the AO-HDS^15,29^ are used. Thus, we anticipate that this thermal management concept based on spin caloritronics will pave the way for energy-efficient electronic and spintronic devices and active thermal devices.

## Methods

### Sample preparation

The [Co/Pt]_*n*_ samples were grown on polished glass substrates by the direct-current magnetron sputtering method from Co and Pt elemental sources at room temperature with a base pressure of <5.0 × 10^−6^ Pa and a working pressure of 0.2 Pa. To obtain a high AO-HDS efficiency, the thicknesses of the Co and Pt layers in the [Co/Pt]_*n*_ multilayer films were optimized to be 0.3 nm and 0.7 nm, respectively (see Supplementary Fig. 1 for details). The [Co/Pt]_*n*_ multilayers are sandwiched between a 3.0-nm-thick Pt capping layer and a 4.3-nm-thick Pt/5.0-nm-thick Ta buffer layer (Fig. 2a). The Pt capping layer is necessary to avoid oxidation, while the bottom Pt/Ta layer improves the adhesion to the substrate and promotes the (111) texture of the [Co/Pt]_*n*_ multilayers for the strong perpendicular magnetic anisotropy^16,20^. For the experiments shown in Fig. 3, the six [Co/Pt]_4_ strips with a width of 0.3 mm were microfabricated from the identical film grown on the same substrate and used for the magnetic domain patterns A–F.

### AO-HDS of magnetization

Before the lock-in thermography measurements, the [Co/Pt]_*n*_ samples were uniformly magnetized by applying an external magnetic field of 20 kOe along the +*z* direction. Then, to form the domain patterns B-F via the AO-HDS, the samples were illuminated with laser pulses with a center wavelength of 514 nm, pulse duration of ~200 fs, and repetition rate of 30 kHz at room temperature. Excitation pulses with a fixed laser power ~2.5 mW were focused on the [Co/Pt]_*n*_ sample surface in a diameter of 12–30 μm, where a corresponding laser fluence range is 0.3–0.7 mJ cm^−2^. Here, the threshold laser fluence to achieve the high AO-HDS efficiency was estimated to be around 0.3 mJ cm^−2^, while too high laser fluence leads to only thermal demagnetization. To ensure the deterministic AO-HDS, we typically applied more than 60,000 laser pulses to the [Co/Pt]_*n*_ samples and swept the laser beam position at a slow velocity of ~3 µm s^−1^. The magnetic domain patterns were subsequently imaged with a magneto-optical Kerr effect microscope.

### Hall measurements

To quantify the magnetization switching ratio triggered by the AO-HDS, we measured the out-of-plane magnetic field *H* dependence of the Hall resistance *R*_H_ in the [Co/Pt]_4_ sample with a Hall cross shape at room temperature. The Hall cross structure with a cross area of 40 × 20 µm^2^ was fabricated by photolithography using a lift-off process and subsequent Ar ion milling. As shown in Fig. 2d, the *H*-*R*_H_ curve shows a rectangular hysteresis loop and the *R*_H_ values remain constant when *H* is greater than the coercive force, indicating that *R*_H_ is dominated by the anomalous Hall effect reflecting the **M** direction. The AO-HDS efficiency was obtained by comparing the *R*_H_ values before and after illuminating the whole Hall cross with **σ**^−^ light at zero field, where the cross was uniformly magnetized by an external field of *H* = +20 kOe before the light illumination. The magnitude of the light-induced *R*_H_ change for the [Co/Pt]_4_ sample was estimated to be ~91% of the *H*-induced *R*_H_ change due to the anomalous Hall effect (Fig. 2d).

### Lock-in thermography

In the lock-in thermography measurements, one inputs a periodic external perturbation, that is, a charge current in our experiments, to a sample and extracts thermal images oscillating with the same frequency as the perturbation^5–9,22^. The first harmonic contribution of the obtained thermal images is transformed into the *A* and *ϕ* images through Fourier analysis. Here, the *A* image shows the distribution of the magnitude of the temperature modulation generated in linear response to the perturbation, while the *ϕ* image gives information about the sign of the temperature modulation as well as the time delay due to thermal diffusion. In general, lock-in thermal images measured at low *f* show temperature distribution in nearly steady states, while those at high *f* show temperature distribution in transient states, where temperature broadening due to thermal diffusion is suppressed (see Supplementary Fig. 3, where the *f* dependence of the lock-in thermal images for our [Co/Pt]_4_ samples is shown). To enhance infrared emissivity and ensure uniform emission properties, the surface of the samples was coated with insulating black ink with the high emissivity (>0.95), commercially available from Japan Sensor Corporation. The infrared radiation intensity *I* detected by an infrared camera is converted into temperature *T* information by measuring the *T* dependence of *I* for the black-ink-coated samples at thermal equilibrium. Here, the *I*-to-*T* conversion for the lock-in thermal images is determined by the differential relation as Δ*T*_1f_ = *dT*/*dI*|_*T*_ Δ*I*_1f_, where Δ*T*_1f_ and Δ*I*_1f_ denote the first harmonic responses of the temperature and infrared radiation intensity, respectively. The lock-in thermography measurements were carried out at room temperature and atmospheric pressure.

## Supplementary information

Supplementary information

## Data Availability

The data that support the findings of this study are available from the corresponding author on reasonable request.
