# Supplementary material for: Magneto-optical painting of heat current
Source: Nat Commun. 2020 Jan 7;11:2. doi: 10.1038/s41467-019-13799-7 (PMC6946696; doi:10.1038/s41467-019-13799-7)
Supplement: Supplementary file 1 — Supplementary information [file 41467_2019_13799_MOESM1_ESM.pdf]

Supplementary Information

Magneto-optical painting of heat current

Wang *et al*.

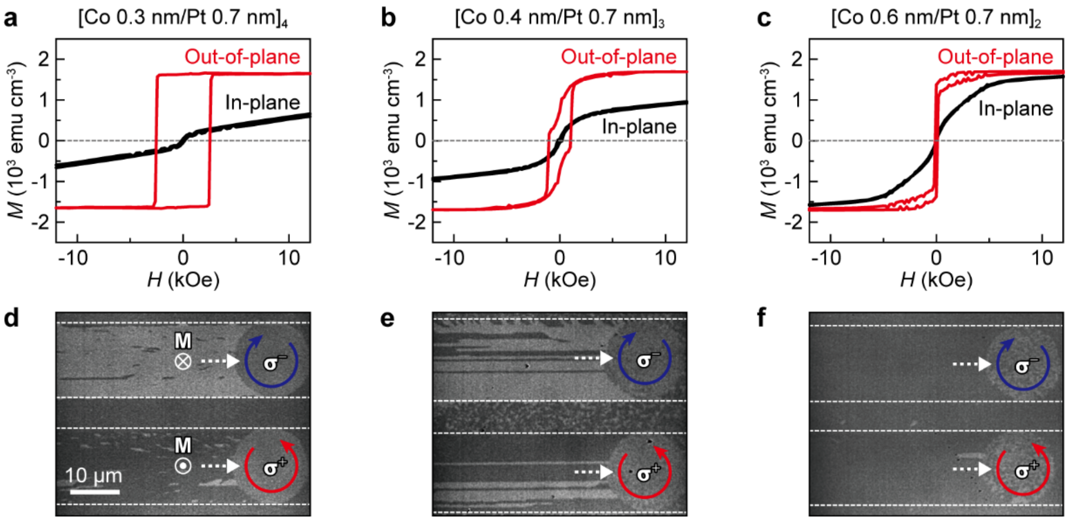

Supplementary Fig. 1 Co layer thickness dependence of magnetic and magneto-optical properties. **a-c** Magnetization *M* curves of the $[Co/Pt]_4$ (**a**), $[Co/Pt]_3$ (**b**), and $[Co/Pt]_2$ (**c**) samples with Co layer thicknesses of 0.3, 0.4, and 0.6 nm, respectively, measured when the magnetic field *H* was applied along the easy axis (out-of-plane direction of the films) and the hard axis (in-plane direction of the films) at room temperature. The total thickness of the Co layers and the thickness of each Pt layer were fixed at 1.2 nm and 0.7 nm, respectively, for all the samples. The samples exhibit the perpendicular magnetic anisotropy, while the out-of-plane coercive field and magnetic anisotropy energy decrease with increasing the Co layer thickness. **d-f** Magneto-optical Kerr effect microscopy images of the $[Co/Pt]_4$ (**d**), $[Co/Pt]_3$ (**e**), and $[Co/Pt]_2$ (**f**) samples illuminated with right (σ^+) and left (σ^-) circularly polarized light at room temperature. **M** denotes the magnetization vector. Before the light illumination, the samples were uniformly magnetized along the upward direction perpendicular to the film plane with an external magnetic field of 20 kOe. The magneto-optical image in **d** indicates the deterministic all-optical helicity-dependent switching (AO-HDS) of magnetization for the $[Co/Pt]_4$ sample with the 0.3-nm-thick Co layers, while the AO-HDS efficiency degrades with increasing the Co layer thickness.

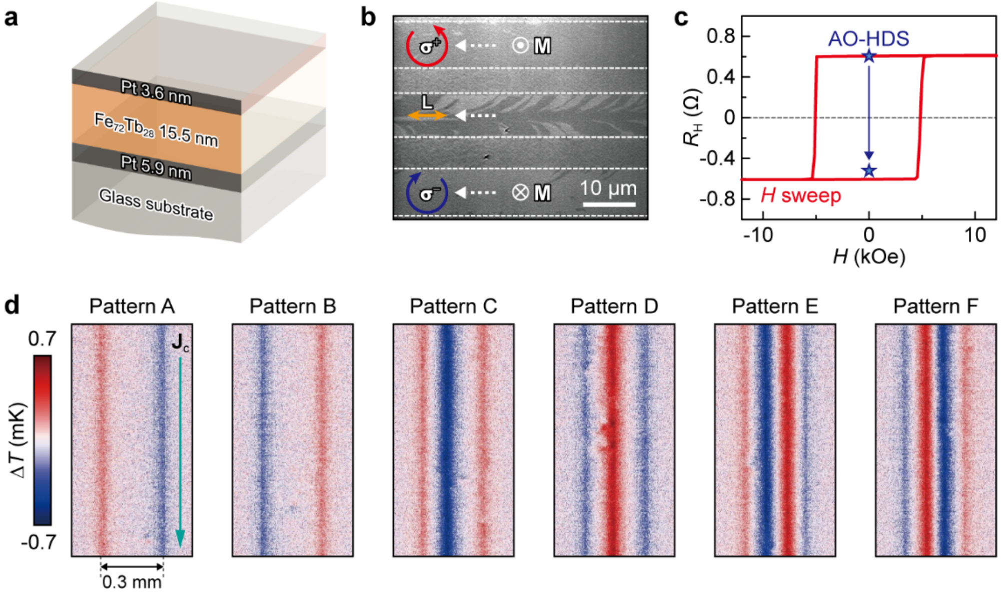

Supplementary Fig. 2 Magneto-optical painting of heat current in ferrimagnetic thin film. **a** Layer configuration of the ferrimagnetic $Fe_{72}Tb_{28}$ alloy film. The 15.5-nm-thick $Fe_{72}Tb_{28}$ layer sandwiched between a 3.6-nm-thick Pt capping layer and a 5.9-nm-thick Pt buffer layer was formed on a glass substrate, where the capping and buffer layers were necessary to avoid oxidation of the $Fe_{72}Tb_{28}$ layer and to improve the adhesion to the substrate, respectively. The $Fe_{72}Tb_{28}$ layer was grown by co-sputtering with Fe and Tb elemental sources, where the source powers controlled the composition. The $Fe_{72}Tb_{28}$ thickness and composition were optimized to show the strong perpendicular magnetic anisotropy and deterministic AO-HDS. **b** Magneto-optical image of the $Fe_{72}Tb_{28}$ sample scanned with circularly ($\boldsymbol{\sigma}^{+}$ and $\boldsymbol{\sigma}^{-}$) and linearly (**L**) polarized light beams at room temperature. **c** Hall resistance R_{H} of the $Fe_{72}Tb_{28}$ sample with a Hall cross shape at room temperature. The Hall cross structure with a cross area of 40×20 μm^2 was fabricated by photolithography using a lift-off process and subsequent Ar ion milling. The red line shows the out-of-plane H dependence of R_{H}. The H-R_{H} curve for the $Fe_{72}Tb_{28}$ sample shows a rectangular hysteresis loop and the R_{H} values remain constant when H is greater than the coercive force (4.8 kOe), indicating that R_{H} is dominated by the anomalous Hall effect reflecting the **M** direction. The blue star data points were measured before and after illuminating the whole Hall cross with $\boldsymbol{\sigma}^{-}$ light at zero field, where the $Fe_{72}Tb_{28}$ sample was uniformly magnetized before the light illumination. **d** ΔT images for the $Fe_{72}Tb_{28}$ strips with various magnetic domain patterns. The $Fe_{72}Tb_{28}$ strips with the patterns A-F exhibit the similar temperature distributions induced by the anomalous Ettingshausen effect (AEE) as those observed for the $[Co/Pt]_4$ strips (compare Supplementary Fig. 2d with Fig. 3g in the main text). Here, the six $Fe_{72}Tb_{28}$ strips with a width of 0.3 mm were microfabricated from the identical film grown on the same substrate.

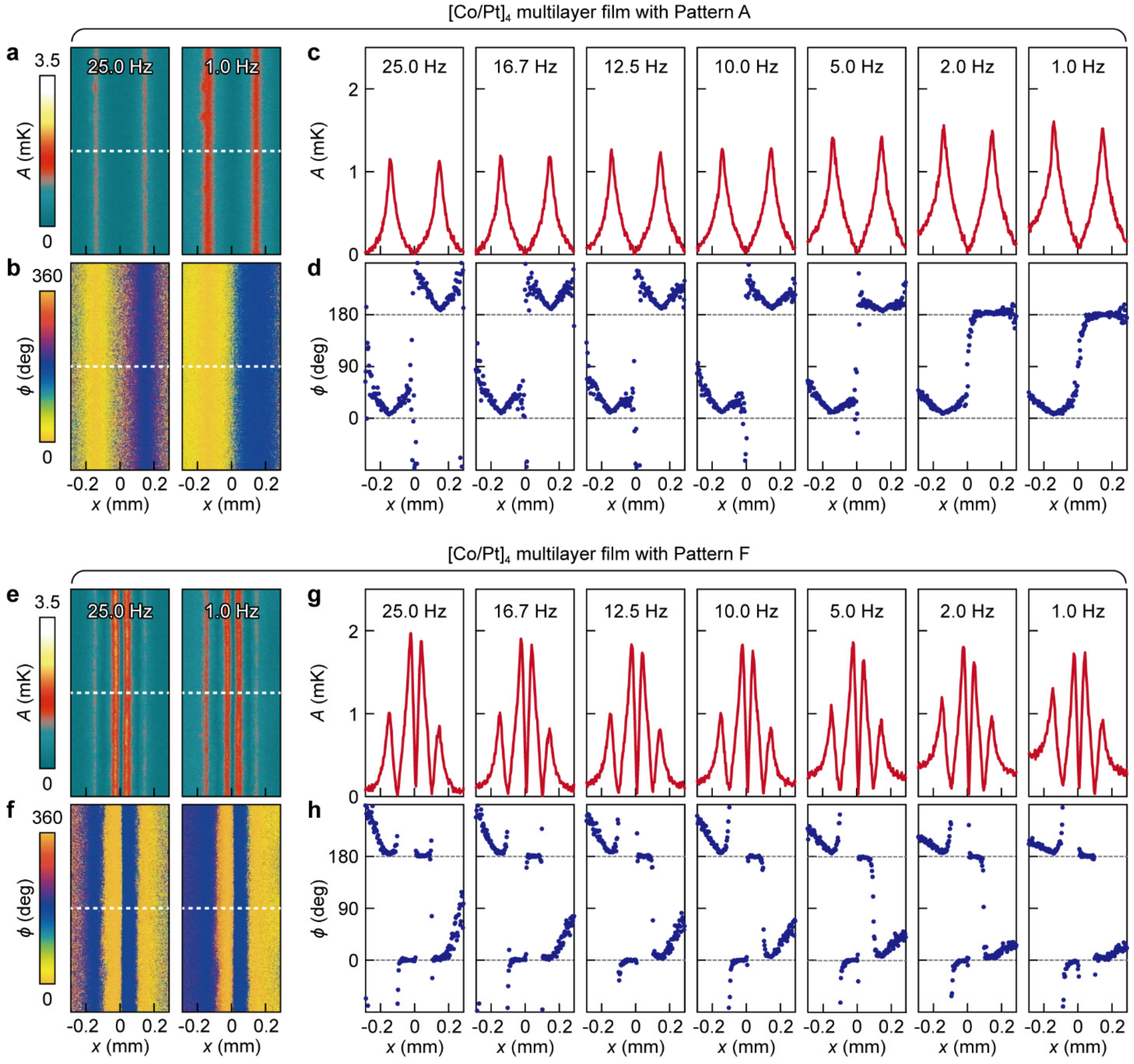

Supplementary Fig. 3 Frequency dependence of temperature modulation. **a**,**b** Lock-in amplitude A (**a**) and phase ϕ (**b**) images for the [Co/Pt]$_4$ strip with the domain pattern A at charge current frequencies of f = 25.0 Hz and 1.0 Hz. These thermal images were obtained under the same condition as that for the data in Fig. 3. **c**,**d** Surface A (**c**) and ϕ (**d**) profiles along the x direction for the [Co/Pt]$_4$ strip with the pattern A for various values of f. The profiles were obtained by averaging 50 x-directional raw profiles along the y direction; the center of the averaged area is marked with white dotted lines in **a** and **b**. The magnitude of the AEE-induced temperature modulation gradually and monotonically increases with decreasing f due to thermal diffusion. **e**,**f** A (**e**) and ϕ (**f**) images for the [Co/Pt]$_4$ strip with the pattern F at f = 25.0 Hz and 1.0 Hz. **g**,**h** Surface A (**g**) and ϕ (**h**) profiles along the x direction for the [Co/Pt]$_4$ strip with the pattern F for various values of f. The f dependence of the AEE-induced temperature modulation for the pattern F is weaker than that for the pattern A, indicating that the temperature modulation for the pattern F reaches a steady state in a shorter time than that for the pattern A. This is because the size of magnetic domains, and the resulting distance between the AEE-induced heat sources and sinks, for the pattern F is smaller than that for the pattern A.
